# Evaluation of Two Parts of *Lithocarpus polystachyus* Rehd. from Different Chinese Areas by Multicomponent Content Determination and Pattern Recognition

**DOI:** 10.1155/2020/8837526

**Published:** 2020-10-24

**Authors:** Min Wei, Yangling Tuo, Ye Zhang, Qi Deng, Cuiying Shi, Xuexian Chen, Xu Zhang

**Affiliations:** ^1^College of Pharmacy, Chengdu University of Traditional Chinese Medicine, Chengdu 611137, China; ^2^People's Hospital of Luxian County, Luzhou 646100, China; ^3^Chengdu Traditional Chinese Medicine Health Care Technology Co. Ltd., Chengdu 610000, China

## Abstract

The purpose of this work is to establish a new method using high-performance liquid chromatography-diode array detection (HPLC-DAD) with chemometrics analysis to determine the content of catechin, isoquercetin, astragalin, phloridzin, trilobatin, and phloretin for one flavanol and five flavonoids, filter out the key compounds, and evaluate the quality of 26 batches of tender leaves and flower spikes of *Lithocarpus polystachyus* Rehd. (LP) from ten areas in China. The result showed that the HPLC-DAD method had excellent performance for accurate quantification analysis. S3 (tender leaf from Lushan, Sichuan) had the highest contents for six measured chemicals with trilobatin content of up to 27.82% in dry weight. S22 (flower spike from Liangping, Chongqing) had the highest content of phloridzin (up to 7.28%). All samples were divided into three types based on spatial distribution using principal component analysis. The result showed that the tender leaves and flower spikes from the same areas had many similar properties, and there were significant differences between the samples from different regions. Furthermore, phloridzin and trilobatin were identified as chemical markers for quality evaluation of two parts with different tender leaves and flower spikes of LP from geographical areas by orthogonal partial least squares discrimination analysis. These results will be helpful to establish an effective and comprehensive evaluation system of the development and utilization of LP resources.

## 1. Introduction

*Lithocarpus polystachyus* Rehd. (LP) belongs to the *Lithocarpus* arbor of the Fagaceae family and is widely distributed in some Asian countries, including China, Thailand, and India [1]. In southern China, tender leaves and flower spikes with sweet tastes have a long history of usage for tea making among ethnic minorities. In addition, the National Health Commission of the People's Republic of China has accepted LP as a new food raw material since 2017. In recent years, LP has been widely used in health care foods, medicine, and other fields. LP is also considered to be an appealing material as bioactive compounds with outstanding pharmacological activities, such as antioxidant, hypolipidemic, hypoglycemic, and anticancer [2–4].

As a new food raw material, LP has huge economic and medical values, which have been artificially cultivated in Hunan, Jiangxi, Chongqing, Sichuan, and other provinces in China [5]. Notably, the products of the tender leaves and flower spikes of LP have appeared in the Chinese market. However, there is no study on the quality divergence or quantification of components of the tender leaves and flower spikes of LP, which is unfavorable to evaluate the quality of LP and standardize market products.

High-performance liquid chromatography (HPLC) is a modern analytical method developed by using high-pressure mobile phase, high-performance stationary phase, and high-sensitivity detector. Compounds with ultraviolet (UV) absorption can be detected by a UV detector. A photodiode-array detector is an optical multichannel UV detector, which emerged in the 1980s. HPLC equipped with diode array detector (DAD), HPLC-DAD, has been widely used in many studies. It can measure the change of absorbance within a certain wavelength range and obtain a 3D spectrum, which can be used for qualitative and quantitative analysis of unknown components. It is widely used in food, medicine, cosmetics analysis, and the formulation of national standards.

In this study, we adopted the HPLC-DAD technology to measure the contents of six known chemicals [6, 7]: catechin, isoquercetin, astragalin, phloridzin, trilobatin, and phloretin with reported pharmacological activity [8–12]. There are no reports of their qualifications. A chemometric method with principal component analysis (PCA) and orthogonal projections to latent structures discriminant analysis (OPLS-DA) was used to find out the chemical markers [13]. The results can provide valuable information for the LP resource explorations as high-quality resources.

## 2. Materials and Methods

### 2.1. Materials and Reagents

**Figure 1:**
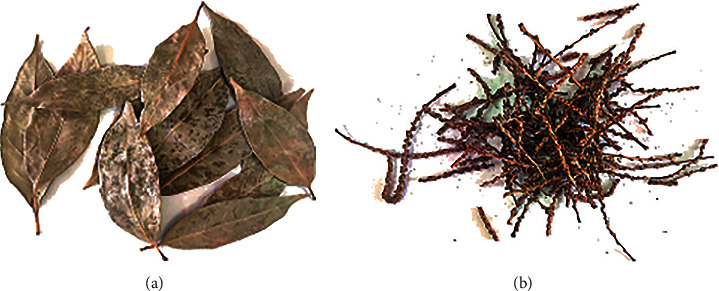
LP sample diagram: (a) LP sample diagram of tender leaves; (b) LP sample diagram of flower spikes.

Twenty-six batches of LP in this experiment were collected from ten different areas in China (see Table 1).  Figure 1 shows the sample diagrams of the tender leaves and flower spikes. The voucher specimens are deposited in the Chengdu University of Traditional Chinese Medicine.

**Figure 2:**
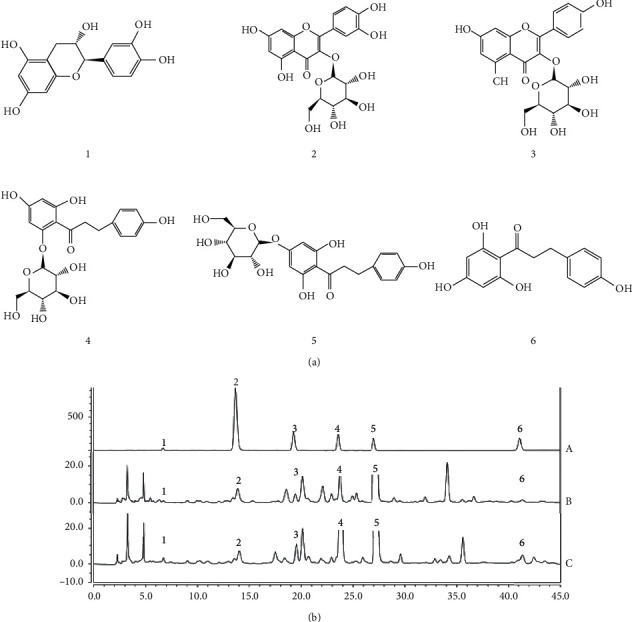
Structures of six chemicals and HPLC chromatograms of two parts of LP. (a) Structure of six chemicals. (1) Catechin, (2) isoquercetin, (3) astragalin, (4) phloridzin, (5) trilobatin, and (6) phloretin. (b) The HPLC chromatogram of six chemicals and LP samples. (A) The HPLC chromatogram of six chemicals. (B) The HPLC chromatogram of tender leaves of S1. (C) The HPLC chromatogram of flower spikes of S1. Peak identification: (1) catechin, (2) isoquercetin, (3) astragalin, (4) phloridzin, (5) trilobatin, and (6) phloretin.

Six standards (see Figure 2(a) were purchased from Chengdu ALFA Biotechnology Co., Ltd. (Chengdu, China) with purity ≥ 98%. Their lot numbers are catechin (AF8030202), isoquercetin (AF9102022), astragalin (AF8062705), phloridzin (AF8060102), trilobatin (AF9091812), and phloretin (AF8030209). Methanol and acetonitrile of chromatographic grade were purchased from Thermo Fisher Scientific of China. Other analysis-grade solvents such as methanol and formic acid were purchased from Chengdu Cologne Chemical Co., Ltd. (Chengdu, China). The aqueous solutions were prepared by deionized water.

### 2.2. Apparatus

Ultimate 3000 HPLC-DAD (Thermo Fisher Scientific); BSA124S electronic balance (1/10,000, German Sartorius company); BT25S electronic balance (1/100,000, German Sartorius company); BT-40A type digital ultrasonic cleaning machine (Chengdu Yayuan Technology Co., Ltd); SL-100 type high-speed crusher (Zhejiang Yongkang Songqing Hardware Factory).

### 2.3. Methods

#### 2.3.1. Preparation of Sample Solution

The samples were crushed and sifted by a sieve with pore sizes of 355 ± 13 *μ*m after drying under 60°C. 0.2 g of the sample power is put into a 100 mL Erlenmeyer flask, and then 25 mL of methanol is added. The total weight of the Erlenmeyer flask with the mixture is recorded. The flasks are put into the ultrasound machine and treated (240 W, 40 kHz) for 25 min. Then, methanol is added to supplement the lost weight. Finally, the mixed solution is filtered via 0.22 *μ*m membrane filters.

#### 2.3.2. Preparation of the Standard Solution

Appropriate amounts of catechin, isoquercetin, astragalin, phloridzin, trilobatin, and phloretin are mixed with a certain volume of methanol to prepare a standard solution, and the concentrations of the six chemicals are 0.30, 0.37, 0.26, 1.12, 2.23, and 0.27 mg/mL, respectively.

#### 2.3.3. HPLC-DAD Conditions

The HPLC-DAD system was carried out with an InerSustainAQ-C18 column (5 *μ*m, 250 × 4.6 mm, GL Sciences). The mobile phase consisted of 0.1% formic acid water (A) and acetonitrile (B) at a flow rate of 0.8 mL/min. The gradient procedures are as follows: 0–10 min, 22% B; 30 min, 35% B; 40 min, 40% B; 40.1–45 min, 22% B. 10 *μ*L of sample solution is injected and detected at 265 nm, and the column temperature is set as 20°C.

### 2.4. HPLC Method Validation

#### 2.4.1. Precision, Repeatability, and Stability

In this study, the intra- and interday precisions are evaluated by injecting S3 sample solution six times a day or once every three days. Meanwhile, six test sample solutions of S3 are parallelly prepared to assess the repeatability of the method. The S3 test sample solution is also separately injected after being placed at ambient temperature for 0, 2, 4, 6, 8, 12, and 24 h for the stability test.

#### 2.4.2. Accuracy

A recovery test is performed to evaluate the accuracy. Six S5 samples with weights of 0.1 g and known contents of different components are weighed. Each portion is added by suitable amounts of the control sample with a weight ratio of 1 : 1. Six test solutions were prepared according to the sample preparation method [14].

#### 2.4.3. Linearity, Limit of Quantification (LOQ), and Limit of Detection (LOD)

The mixed standard solution is diluted by 0, 2, 20, 40, 200, and 250 times with methanol, respectively, for the six test solutions. They are measured according to the HPLC-DAD conditions; the concentrations of the reference (mg/mL) are taken as the abscissa (*X*) and the peak areas as the ordinate (*Y*) for linear regression. LOQ is calculated with the signal-to-noise ratio (SNR) as 10 : 1 for all analytes, and LOD is calculated with the SNR as 3 : 1.

### 2.5. Sample Determination

26 samples of LP are prepared as the test solutions (*n* = 3) by the sample preparation method, and, simultaneously, the contents of catechin, isoquercetin, astragalin, phloridzin, trilobatin, and phloretin are determined under the HPLC-DAD conditions.

### 2.6. Pattern Recognition Analysis

The content data of the six target chemicals are imported into Simca-p 14.1 software and analyzed by PCA followed by OPLS-DA. PCA is an unsupervised pattern recognition to reduce the dimensionality of a data set by a linear transformation. It is used to find groupings and clusters of interrelated samples to establish a relationship between samples and variables. OPLS-DA is a supervised algorithm and is commonly used to analyze chemical variations between inner and outer groups and identify potential markers [13].

## 3. Results and Discussion

### 3.1. Condition Optimization

#### 3.1.1. Optimization of LP Extraction Conditions

**Figure 3:**
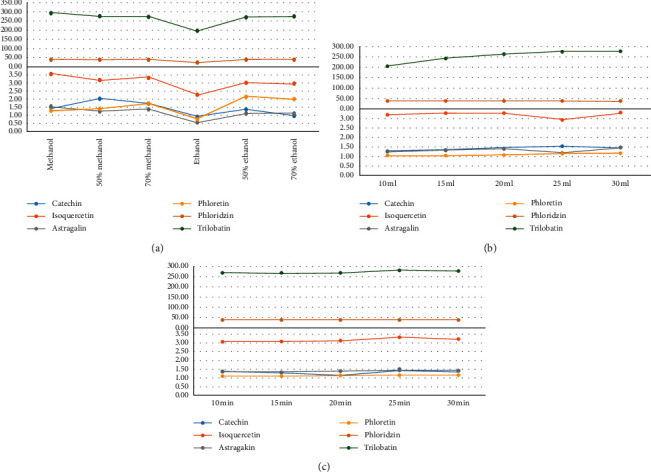
Optimization of LP extraction conditions. (a) Solvent type (methanol, 50% methanol, 70% methanol, ethanol, 50% ethanol, and 70% ethanol). (b) Solvent volume (10, 15, 20, 25, and 30 mL). (c) Ultrasonic time (10, 15, 20, 25, and 30 min).

The single factor experiment method is used to examine the effects of the type of solvent (methanol, 50% methanol, 70% methanol, ethanol, 50% ethanol, and 70% ethanol), the volume of added solvent (10, 15, 20, 25, and 30 mL), and the ultrasonic time (10, 15, 20, 25, and 30 min) on the targeted component extractions.  Figure 3 shows the results. Ethanol has the lowest dissolution for the target components, but the extraction capacity of methanol with 70% ethanol is much better. Considering the low contents of catechin, isoquercetin, and astragalin in LP, methanol is selected as the extraction solvent. The extraction amount of the components increases with the increase of the solvent volume and ultrasonic time but tends to slow down after increasing to a certain extent. Therefore, 25 mL methanol and 25 min for ultrasonication are selected as the extraction conditions.

#### 3.1.2. Optimization of Chromatographic Conditions

**Figure 4:**
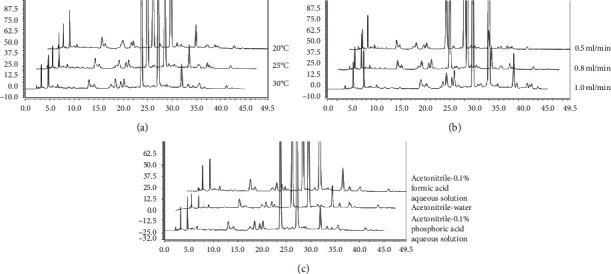
Optimization of chromatographic conditions. (a) Column temperature (20, 25, and 30°C). (b) Flow rate (0.5, 0.8, and 1.0 mL/min). (c) Mobile phase (acetonitrile-0.1% formic acid aqueous solution, acetonitrile-water, and acetonitrile-0.1% phosphoric acid aqueous solution).

The retention time is appropriate and the peak shape is symmetrical when acetonitrile is used as the mobile phase. Therefore, column temperature (20, 25, and 35°C), flow rate (0.5, 0.8, and 1.0 mL/min), and concentration of mobile phase (acetonitrile-0.1% formic acid aqueous solution, acetonitrile-water, and acetonitrile-0.1% phosphoric acid aqueous solution) are examined as optimization conditions. The results show that the best separation effect is obtained when the column temperature, flow rate, and concentration of mobile phase are 20°C, 0.8 ml/min, and acetonitrile-0.1% formic acid solution (see Figure 4).

### 3.2. Adaptability of HPLC-DAD Condition

Figure 2(b) displays the chromatograms of mixed standard solution and LP sample solutions. The separating degrees of target components in the samples are greater than 1.5. In addition, the baseline is stable and the number of theoretical plates is more than 3000 for the six target chemicals.

### 3.3. HPLC Method Validation

#### 3.3.1. Precision, Repeatability, and Stability

From Table 2, the relative standard deviations (RSDs) of peak areas of the six chemicals range from 0.13% to 1.53% in the precision test, which indicates excellent precision of the instrument. The RSDs of chemicals contents in the repeatability test are all less than 2.16%, thereby indicating satisfactory repeatability of the method. The test solution is stable within 24 h with the fact that the RSDs of peak areas of chemicals are all less than 1.91%.

#### 3.3.2. Accuracy

The calculation formula of the recovery experiment

(1) $$\begin{array}{c} R\left(\%\right)=\frac{M_{\text{F}}-M_{\text{K}}}{M_{\text{A}}}\times 100\% \end{array}$$

is (*M*_F_ : found, *M*_K_ : known, *M*_A_ : added). The equation is suitable for content analysis when the percentage recovery of chemicals ranges from 95.28% to 104.94%, and the RSDs are less than 3% (see Table 3).

#### 3.3.3. Linearity, LOQ, and LOD

There is a linear relationship between the peak areas and the compound concentrations over a wide concentration range with the coefficient of correlation (*r*) over 0.9992 (see Table 4). Table 4 also shows the LOQ and LOD.

### 3.4. Sample Analysis

In recent years, flavonoids in plants have attracted much more attention. Their activities, such as hypoglycemic, compressive, antibacterial, and anticancer activities, have been confirmed. Most of the flavonoids in plants form glycosides from sugars through glycosidic bonds. Isoquercetin, astragalin, phloridzin, and trilobatin are linked with one glucose group to form glycosides except for catechin and phloretin. The main chemical components of LP are flavonoid glycosides. Phloridzin and trilobatin belong to dihydrochalcone glucoside compounds, and they are also very hot research components in LP recently [8, 9]. At the same time, trilobatin is considered as the main source of sweetness in LP, as its sweetness is 300 times higher than that of sucrose [15]. The contents of catechin, isoquercetin, astragalin, phloridzin, trilobatin, and phloretin in the tender leaves (S1–S16) and the flower spikes (S17–S26) of LP samples from different areas are determined by the HPLC-DAD method (see Table 5). The content ranges of the six chemicals in the 26 samples are 0.39–1.57 mg/g (catechin), 0.49–3.20 mg/g, (isoquercetin), 0.41–2.39 mg/g (astragalin), 4.89–72.81 mg/g (phloridzin), 12.40–278.15 mg/g (trilobatin), and 0.36–4.98 mg/g (phloretin). The average contents are 0.90, 1.41, 1.20, 37.00, 140.86, and 1.18 mg/g. It is noticeable that the content of trilobatin was the most in all components for the 26 samples except for S5, S22, and S23. Furthermore, the tender leaf of S3 (tender leaf from Lushan, Sichuan) has the highest value in weight, 27.82%. S22 (flower spike of Liangping, Chongqing) has the highest content of phloridzin up to 7.28%. In 26 batches of LP samples, the average content of phloridzin in the tender leaves is 24.77 mg/g, which is lower than that in the flower spikes (56.57 mg/g). The average content of trilobatin in the tender leaves is 146.26 mg/g, which is higher than that in the flower spikes (132.228 mg/g), seeing that the content of phloridzin in the tender leaves and flower spikes changed more than that in trilobatin. Asides different parts of LP, the factors causing the differences may also be related to the growing environment and harvest time of LP. In general, it is difficult to identify quality differences between the tender leaves and flower spikes of LP from these results. Therefore, it is necessary to search for potential markers that can help to control the quality of the tender leaves and flower spikes of LP to provide references for the study of LP resources and guide the rational development and utilization of LP resources.

### 3.5. Pattern Recognition Analysis

#### 3.5.1. PCA

**Figure 5:**
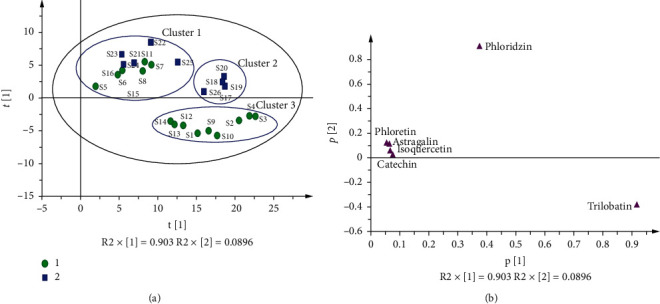
PCA analysis of the contents of six chemicals of LP samples. (a) PCA score plot shows distribution of samples into three clusters, series ((1) tender leaf; (2) flower spike). (b) Loading plot shows distribution of variables.

Three principal components were extracted to deliver 99.8% (goodness of fit: R2X (cum) = 0.998) of the original data set, and predictive ability of the model was 90.9% (Q2 (cum) = 0.909). 26 samples are divided into three clusters in the score plot (see Figure 5(a)). S5–S8, S11, S15–S16, and S21–S24 belong to cluster 1, showing many similarities in the tender leaves and flower spikes from the same areas. The flower spikes and tender leaves gathered in cluster 2 (flower spikes of S17–S20 and S26) and cluster 3 (tender leaves of S1–S4, S9, and S10–S14) show that the tender leaves and flower spikes from different areas can be distinguished by component content. In addition, the score and loading plots (see Figure 5(b)) are used to explain the relationship between principal components and samples, and the sample is heavily influenced by the related component when they are from the same direction. Therefore, the content of phloridzin in S22 (flower spike of Liangping, Chongqing) is the highest, and the content of trilobatin is also the highest in S3 (tender leaf of Lushan, Sichuan). However, S5 has the lowest contents of phloridzin and trilobatin.

#### 3.5.2. OPLS-DA

**Figure 6:**
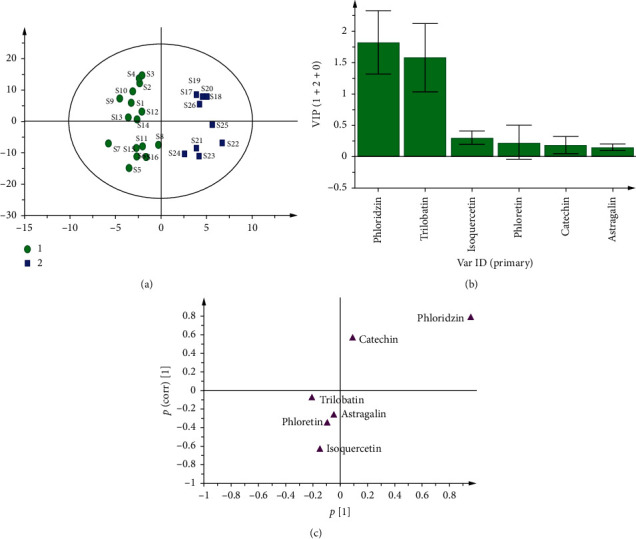
OPLS-DA analysis of the contents of six chemicals of LP samples. (a) OPLS-DA score plot shows distribution of samples into two clusters, series ((1) tender leaf; (2) flower spike). (b) Variable importance for the projection (VIP). (c) An S-plot highlights variables.

Based on PCA grouping, OPLS-DA analysis is conducted after Pareto scaling to further investigate the variables which are responsible for cluster classification of LP. OPLS-DA resulted in a (1 + 2 + 0) component with excellent model parameters: R2X (cum) = 99.4%, R2Y (cum) = 90.4%, and Q2 (cum) = 84.8%. From the score plot (see Figure 6(a)), the tender leaves and flower spikes are clearly distributed in both positive and negative directions in the model, hence indicating that there are significant differences in chemical composition. Meanwhile, there is obvious intrapopulation variation between the tender leaves and flower spikes, thus indicating the differences of their areas. In addition, the variable importance in projection (VIP) and S-plot are effective biomarkers for identifying the factors that lead to the grouping of samples in the OPLS-DA model. The VIP values of phloridzin and trilobatin are more than 1 (see Figure 6(b)), and phloridzin and trilobatin are scattered in both ends and far away from the center point in Figure 6(c), which means that phloridzin and trilobatin are potential markers [16]. Furthermore, in terms of composition distribution, phloridzin highly correlates with the flower spikes, and trilobatin has a large contribution to the tender leaves.

## 4. Conclusion

This study established an HPLC-DAD method with excellent parameters of separation and method validation for the content determination of catechin, isoquercetin, astragaloside, phloridzin, trilobatin, and phloretin for 26 batches of tender leaves and flower spikes of LP. The results showed that there are substantial contents of trilobatin in the tender leaves and flower spikes. Pattern recognition analysis was used to analyze the contents of six components of the total samples. The component contents of the tender leaves and flower spikes are significantly different in general. In addition, phloridzin and trilobatin are potential markers to distinguish the tender leaves and flower spikes. These results contribute to the development and utilization of the LP resources.

## Figures and Tables

**Table 1:** Sample information.

Number	Part	Location
S1	Tender leaf	Lushan, Sichuan
S2	Tender leaf	Lushan, Sichuan
S3	Tender leaf	Lushan, Sichuan
S4	Tender leaf	Lushan, Sichuan
S5	Tender leaf	Dazhu, Sichuan
S6	Tender leaf	Liangqing, Chongqing
S7	Tender leaf	Longsheng, Guangxi
S8	Tender leaf	Dazhou, Sichuan
S9	Tender leaf	Dongkou, Hunan
S10	Tender leaf	Zhijiang, Hunan
S11	Tender leaf	Anfu, Jiangxi
S12	Tender leaf	Guizhou
S13	Tender leaf	Guizhou

**Table 2:** The results of precision, repeatability, and stability.

Compound	Precision		Repeatability		Stability
	Intraday RSD (%)	Interday RSD (%)	Average contents (mg/g)	RSD (%)	RSD (%)
Catechin	1.12	1.53	1.34	1.16	1.39
Isoquercetin	1.17	1.08	2.58	1.17	1.66
Astragalin	0.64	0.82	1.09	0.89	1.46
Phloridzin	0.27	0.35	35.84	0.89	0.10
Trilobatin	0.13	0.27	273.66	0.80	0.11
Phloretin	0.96	1.26	1.14	2.16	1.91

**Table 3:** Results of the recovery test.

Compound	Known (mg)	Added (mg)	Found (mg)	Recovery (%)	Average recovery (%)	RSD (%)
Catechin	0.05	0.04	0.09	98.48	101.57	2.02
	0.05	0.04	0.09	103.95		
	0.05	0.04	0.09	102.22		
	0.04	0.04	0.08	100.59		
	0.05	0.04	0.09	100.68		
	0.04	0.04	0.09	103.49		

Isoquercetin	0.12	0.10	0.23	102.49	102.31	2.31
	0.12	0.10	0.22	97.97		
	0.12	0.10	0.23	104.35		
	0.11	0.10	0.22	104.47		
	0.12	0.10	0.22	101.96		
	0.11	0.10	0.22	102.61		

Astragalin	0.18	0.16	0.33	99.80	99.22	2.33
	0.17	0.16	0.33	97.46		
	0.17	0.16	0.33	99.86		
	0.17	0.16	0.32	96.57		
	0.17	0.16	0.34	103.13		
	0.17	0.16	0.32	98.53		

Phloridzin	1.37	1.21	2.55	97.91	101.82	2.31
	1.34	1.21	2.55	100.55		
	1.33	1.21	2.59	104.12		
	1.29	1.21	2.52	102.37		
	1.35	1.21	2.58	101.90		
	1.30	1.21	2.55	104.08		

Trilobatin	1.33	1.24	2.63	104.94	103.08	1.59
	1.29	1.24	2.58	104.08		
	1.29	1.24	2.57	103.43		
	1.25	1.24	2.50	100.92		
	1.31	1.24	2.56	101.21		
	1.26	1.24	2.55	103.91		

Phloretin	0.09	0.08	0.17	99.88	99.45	2.83
	0.09	0.08	0.17	101.54		
	0.09	0.08	0.17	102.38		
	0.08	0.08	0.16	95.28		
	0.09	0.08	0.17	100.84		
	0.08	0.08	0.16	96. 5		

**Table 4:** Linear relationship, LOQ, and LOD.

Compound	Regression equation	*r*	Linear range (*μ*g·mL^−1^)	LOQ (*μ*g·mL^−1^)	LOD (*μ*g·mL^−1^)
Catechin	*Y* = 36.41*X* − 0.0462	0.9994	1.20–300.00	0.34	0.12
Isoquercetin	*Y* = 282.44*X* − 0.5162	0.9993	1.46–36.50	0.15	0.05
Astragalin	*Y* = 255.80*X* − 0.2825	0.9992	1.04–260.00	0.10	0.04
Phloridzin	*Y* = 115.84*X* − 0.3612	0.9995	4.50–1120.00	0.16	0.06
Trilobatin	*Y* = 177.93*X* + 0.2167	0.9999	8.90–2225.00	0.16	0.05
Phloretin	*Y* = 188.99*X* − 0.1707	0.9994	1.06–265.00	0.11	0.05

**Table 5:** Content results of 26 batches of six chemicals (mg/g, *n* = 3).

Number	Catechin		Isoquercetin		Astragalin		Phloridzin		Trilobatin		Phloretin		Total	Total
	Content	Rank	Content	Rank	Content	Rank	Content	Rank	Content	Rank	Content	Rank	Contents	Rank
S1	0.62 ± 0.04	19	1.19 ± 0.07	11	0.70 ± 0.03	21	4.89 ± 0.15	26	204.33 ± 5.80	8	0.42 ± 0.02	23	212.15	11
S2	1.22 ± 0.01	7	2.62 ± 0.06	4	1.18 ± 0.02	14	29.01 ± 0.26	18	257.25 ± 5.26	3	0.97 ± 0.05	11	292.25	3
S3	1.36 ± 0.01	6	3.20 ± 0.02	1	1.40 ± 0.01	9	38.20 ± 0.20	14	278.15 ± 0.79	1	1.17 ± 0.01	7	323.48	1
S4	1.22 ± 0.00	7	3.10 ± 0.00	2	1.36 ± 0.00	11	36.20 ± 0.58	15	270.34 ± 0.72	2	1.25 ± 0.02	5	313.47	2
S5	0.44 ± 0.03	24	1.12 ± 0.04	12	1.65 ± 0.02	4	12.80 ± 0.18	20	12.40 ± 0.54	26	0.82 ± 0.07	17	29.23	26
S6	0.39 ± 0.00	25	2.02 ± 0.00	6	2.39 ± 0.01	1	36.19 ± 0.24	16	41.87 ± 0.62	22	1.36 ± 0.11	3	84.22	24
S7	0.48 ± 0.02	23	3.02 ± 0.03	3	2.27 ± 0.02	2	48.74 ± 0.41	10	82.03 ± 1.08	16	4.98 ± 0.26	1	141.52	16
S8	0.56 ± 0.03	20	1.25 ± 0.02	10	1.48 ± 0.02	7	42.84 ± 0.86	7	73.32 ± 1.16	17	1.97 ± 0.15	11	131.42	18
S9	0.74 ± 0.03	15	1.91 ± 0.02	7	1.18 ± 0.01	14	10.06 ± 0.18	21	218.16 ± 0.54	5	1.00 ± 0.01	10	233.04	9
S10	0.82 ± 0.05	13	1.06 ± 0.05	15	0.50 ± 0.02	23	8.69 ± 1.23	22	236.47 ± 9.49	4	1.25 ± 0.14	5	248.79	8
S11	N/A	26	1.10 ± 0.01	13	2.08 ± 0.00	3	49.84 ± 0.35	9	69.56 ± 1.04	18	4.16 ± 0.06	2	126.74	19
S12	0.89 ± 0.01	12	1.02 ± 0.01	17	0.41 ± 0.00	26	7.46 ± 0.22	23	176. 90 ± 2.48	12	0.36 ± 0.00	25	187.04	13
S13	0.50 ± 0.03	22	1.35 ± 0.02	9	0.87 ± 0.01	19	5.26 ± 0.36	25	161. 42 ± 1.83	13	0.38 ± 0.02	24	169.78	14
S14	0.72 ± 0.03	16	1.10 ± 0.01	13	0.43 ± 0.00	24	6.65 ± 0.05	24	153. 84 ± 1.03	14	0.52 ± 0.13	22	163.26	15
S15	0.90 ± 0.03	11	2.07 ± 0.01	5	1.54 ± 0.01	6	28.27 ± 0.24	19	65.90 ± 0.27	19	1.36 ± 0.11	3	100.04	21
S16	0.56 ± 0.05	20	1.73 ± 0.08	8	1.61 ± 0.08	5	31.24 ± 1.58	17	38.21 ± 1.71	24	0.94 ± 0.02	13	74.29	25
S17	1.57 ± 0.05	1	1.01 ± 0.02	18	1.37 ± 0.05	10	56.12 ± 0.92	6	211.15 ± 3.06	7	0.77 ± 0.07	18	271.99	5
S18	1.42 ± 0.08	4	0.96 ± 0.02	19	1.28 ± 0.02	13	60.21 ± 1.79	4	203.50 ± 4.21	9	0.77 ± 0.09	18	268.4	7
S19	1.56 ± 0.06	2	1.03 ± 0.02	16	1.42 ± 0.04	8	57.09 ± 0.66	5	211.72 ± 2.29	6	0.83 ± 0.05	16	273.65	4
S20	1.39 ± 0.02	5	0.96 ± 0.02	19	1.34 ± 0.03	12	64.94 ± 0.24	2	202.25 ± 0.78	10	0.87 ± 0.02	15	271.75	6
S21	1.50 ± 0.05	3	0.64 ± 0.01	23	1.04 ± 0.03	17	48.52 ± 0.41	11	54.89 ± 1.06	21	0.54 ± 0.04	21	107.13	20
S22	1.22 ± 0.03	7	0.80 ± 0.04	21	1. 06 ± 0.06	16	72.81 ± 2.25	1	64.60 ± 2.75	20	0.92 ± 0.02	14	141.41	17
S23	0.70 ± 0.01	17	0.49 ± 0.00	26	0.65 ± 0.00	22	53.24 ± 0.91	8	29.34 ± 0.94	25	1.11 ± 0.04	8	85.53	23
S24	0.80 ± 0.07	14	0.56 ± 0.02	24	0.75 ± 0.04	20	43.43 ± 1.58	13	39.82 ± 1.26	23	1.08 ± 0.02	9	86.44	22
S25	0.63 ± 0.01	18	0.72 ± 0.03	22	0.88 ± 0.03	18	63.66 ± 0.80	3	121.38 ± 1.06	15	0.64 ± 0.04	20	187.91	12
S26	1.09 ± 0.07	10	0.56 ± 0.03	24	0.42 ± 0.03	25	45.71 ± 3.47	12	183.63 ± 2.68	11	0.36 ± 0.00	25	231.77	10

Note: data presented as mean ± SD. “N/A” = under LOD.

## Data Availability

The data are generated and analyzed gradually in the process of the experiment. All the authors guarantee the correctness of the experimental data.

## References

[1] Li Y., Guo W., He P., Yu L. (2019). The complete chloroplast genome of sweet tea (*Lithocarpus polystachyus*). *Mitochondrial DNA Part B*.

[2] Hou S.-z., Chen S.-x., Huang S. (2011). The hypoglycemic activity of *Lithocarpus polystachyus* Rehd. leaves in the experimental hyperglycemic rats. *Journal of Ethnopharmacology*.

[3] Hou S.-z., Xu S.-j., Jiang D.-x. (2012). Effect of the flavonoid fraction of *Lithocarpus polystachyus* Rehd. on spontaneously hypertensive and normotensive rats. *Journal of Ethnopharmacology*.

[4] Lin C., Wang L., Wang H. (2014). *Lithocarpus polystachyus* Rehd leaf aqueous extract inhibits human breast cancer growth in vitro and in vivo. *Nutrition and Cancer*.

[5] Wang K., Li B. C., Ling W. H., Li K. X. (2019). Analysis and evaluation on main economic traits and active constituents of thirty *Lithocarpus ploystachyus* Rehd. provenances. *Southwest China Journal of Agricultural Sciences*.

[6] Li X., Zhao Y., Hou S. (2014). Identification of the bioactive components of orally administered *Lithocarpus polystachyus* Rehd and their metabolites in rats by liquid chromatography coupled to LTQ orbitrap mass spectrometry. *Journal of Chromatography B*.

[7] Wang M., Liu X., Zhang Z., Yu J., Liu J., Wu Y. (2020). Phytochemicals and bioactive analysis of different sweet tea (*Lithocarpus litseifolius* [Hance] Chun) varieties. *Journal of Food Biochemistry*.

[8] Gao J., Chen N., Li N. (2020). Neuroprotective effects of trilobatin, a novel naturally occurring Sirt3 aganist from *Lithocarpus polystachyus* Rehd., mitigate cerebral ischemia/reperfusion injury: involvement of TLR4/NF-*κ*B and nrf2/keap-1 signaling. *Antioxidants & Redox Signaling*.

[9] Kumar S., Sinha K., Sharma R., Purohit R., Padwad Y. (2019). Phloretin and phloridzin improve insulin sensitivity and enhance glucose uptake by subverting PPAR*γ*/Cdk5 interaction in differentiated adipocytes. *Experimental Cell Research*.

[10] Luo C., Xu X., Wei X. (2019). Natural medicines for the treatment of fatigue: bioactive components, pharmacology, and mechanisms. *Pharmacological Research*.

[11] Reygaert W. C. (2018). Green tea catechins: their use in treating and preventing infectious diseases. *BioMed Research International*.

[12] Zheng D., Liu D., Liu N., Kuang Y., Tai Q. (2019). Astragalin reduces lipopolysaccharide-induced acute lung injury in rats via induction of heme oxygenase-1. *Archives of Pharmacal Research*.

[13] Nsuala B. N., Kamatou G. P., Sandasi M., Enslin G., Viljoen A. (2017). Variation in essential oil composition of Leonotis leonurus, an important medicinal plant in South Africa. *Biochemical Systematics and Ecology*.

[14] Zhang J., Chen L., Qiu J. (2020). Simultaneous determination of six chromones in saposhnikoviae radix via quantitative analysis of multicomponents by single marker. *Journal of Analytical Methods in Chemistry*.

[15] Chen Z.-H., Zhang R.-J., Wu J., Zhao W.-M. (2009). New dihydrochalcone glycosides fromLithocarpus litseifoliusand the phenomenon of C-H ⟶ C-D exchange observed in NMR spectra of phenolic components. *Journal of Asian Natural Products Research*.

[16] Wang X., Ma C., Yang P. (2020). Integrated HPLC fingerprinting and multivariate analysis differentiates between wild and cultivated *Hedyotis diffusa* willd. *Industrial Crops and Products*.

